# Effect of Natural Inhibitors on the Corrosion Properties of Grade 2 Titanium Alloy

**DOI:** 10.3390/ma17215202

**Published:** 2024-10-25

**Authors:** Mehrdad Faraji, Luca Pezzato, Arshad Yazdanpanah, Giacomo Nardi, Mojtaba Esmailzadeh, Irene Calliari

**Affiliations:** 1 Department of Industrial Engineering, University of Padova, Via Marzolo 9, 35131 Padova, Italy; mehrdad.faraji@studenti.unipd.it (M.F.); arshad.yazdanpanah@phd.unipd.it (A.Y.); giacomo.nardi.1@studenti.unipd.it (G.N.); irene.calliari@unipd.it (I.C.); 2 National Institute for Nuclear Physics (INFN), Padova Division, Via Marzolo 8, 35131 Padova, Italy; 3 Institute of Condensed Matter Chemistry and Energy Technologies (ICMATE), National Research Council of Italy, C.so Stati Uniti 4, 35127 Padova, Italy; 4 Department of Mechanical Engineering, Persian Gulf University, Bushehr 75169-13817, Iran; m.esmaeilzade@pgu.ac.ir

**Keywords:** natural inhibitor, corrosion, electrochemical study, titanium, molecular dynamic simulation

## Abstract

This study investigates the effects of natural inhibitors (pomegranate, algae, and tomato extracts) on the corrosion resistance of titanium (grade 2). To deepen understanding the inhibition mechanism, Molecular Dynamic (MD) and Monte Carlo (MC) simulations were employed to analyze adsorption behaviors and identify optimal adsorption sites on titanium oxide (TiO_2_) surfaces for compounds within the inhibitors. Results indicate non-flat adsorption orientations, with pomegranate peel extract components showing superior inhibition capabilities, attributed to the formation of strong O-H chemical bonds with the TiO_2_ surface. In the experimental part of the study Electrochemical Impedance Spectroscopy (EIS) and Potentiodynamic Polarization (PDP) were conducted. Two electrolytes were tested: a solution 3.5% NaCl and a solution 0.5 M NaOH. All the tests were performed with 5% of inhibitor and with the reference solution. Also, inhibition efficiency was calculated on the base of PDP tests. The study found that pomegranate extract can act as a good corrosion inhibitor for titanium alloy in aqueous solutions 0.5 M NaOH. This was demonstrated by the increase in the corrosion potential and impedance modulus and decrease in the corrosion current density after the addition of pomegranate extract to the solution. However, in a 3.5% NaCl solution, the efficacy of pomegranate extract was less pronounced, probably due to the high aggressivity of the electrolyte. Tomato and algae extract have instead shown very low inhibition effects in all the tested conditions.

## 1. Introduction

Corrosion is one of the most destructive phenomena that affects many industrial sectors, such as the marine construction, oil and gas and automotive sectors [1,2,3,4]. Numerous studies have been conducted via different inhibitors to prevent harmful effects of corrosive processes [5,6]. In particular the interest in green inhibitors as eco-friendly materials has currently grown to address corrosion problems [7]. In recent years, several research have been conducted on employing plant and fruit extracts as natural inhibitors, highlighting them as environmentally friendly alternatives to traditional inhibitors for preventing metal corrosion. The advantages of using these green inhibitors are manifold. Firstly, they effectively safeguard metals from corrosion while preserving ecological equilibrium. Secondly, being derived from renewable, biodegradable, and eco-friendly resources, they contribute to minimizing environmental contamination. Compared to traditional inhibitors, they are not only more economical but are also capable of being recycled. Therefore, integrating green inhibitors into metal corrosion prevention strategies is advocated as a responsible, affordable, and eco-conscious approach [8,9,10,11]. This is demonstrated also by the fact that the interest of the scientific research on the use of natural inhibitors is growing in the recent years: for example, Okutan et al. [12] use walnut extract for the inhibition of corrosion of mild steel, whereas other authors use natural inhibitors for the protection of mild steel in concrete [13,14].

Titanium, renowned for its lightweight yet robust properties, finds extensive application in various industries, particularly in the biomedical field and in applications in marine environments. A protective oxide layer on titanium’s surface provides generally excellent corrosion resistance, but the oxide layer may dissolve in certain environments, such as chloride solutions, then producing corrosion phenomena [15]. In detail, grade 2 titanium possesses excellent corrosion properties, thanks to the possibility to form a thin compact protective oxide film, but in de-aerated conditions or in low pH reducing environments, instability of the passive film compromises the metal integrity [16]. Consequently, despite its numerous advantages, corrosion remains a significant challenge in the utilization of titanium in corrosive media. Extensive research is underway to enhance the service life and performance of this metal under such conditions [17].

Numerous compounds derived from plants act as corrosion inhibitors, with their effectiveness often linked to the presence of phytochemicals like tannins, polyphenols, flavonoids, phlobatannins, anthraquinones, saponins, alkaloids, and organic sugars. These substances can bind to metal surfaces, preventing cathodic, anodic, or both types of reactions from occurring [18,19,20]. Research has explored the potential of different fruit components, including those found in peaches, apricots, and pomegranates, as corrosion inhibitors [21,22]. Recently, pomegranate fruit has garnered attention for its applications beyond the medical and food industries, extending into engineering contexts [23,24,25].

The mature section of the pomegranate fruit is rich in sugars, acids, vitamins, polyphenols, polysaccharides and crucial nutrients [23]. It is recognized for its medicinal benefits, including antioxidant, antibacterial and inhibitory effects, which are attributed to its content of anthocyanins like pelargonidin, delphinidin and cyaniding, along with other phenolic compounds such as hydrolysable tannins including punicalin, punicalagin, gallagic, pedunculagin, ellagic acid and organic acids [21,26,27,28,29,30,31,32]. El-Etre et al. [33] showed that lawsonia extract works well to stop steel from rusting in hydrochloric acid, proving natural products can help with rust. Raja et al. [34] studied how Solanum tuberosum (potato) peel extract stops mild steel from rusting in sulfuric acid. They pointed out that using leftover farm material to stop rust is cost effective and environmentally friendly. Research has shown that plant extracts, such as those from Artemisia vulgaris, can be highly effective, achieving up to 93% inhibition efficiency in certain conditions like acidic environments [35]. Additionally, studies have explored the use of various natural substances such as tannins, flavonoids and alkaloids, which form protective layers on metal surfaces and inhibit corrosion through adsorption mechanisms [36]. The use of natural inhibitors is particularly appealing due to their renewable and biodegradable nature, aligning with environmental regulations aimed at reducing toxicity in industrial applications [35].

This research aims to examine the impact of pomegranate extract as a natural inhibitor on the corrosion of Titanium alloy (grade 2), evaluated in two different aqueous solutions: 3.5% NaCl and 0.5 M NaOH. Furthermore, two additional natural inhibitors, tomato and algae extracts, were also assessed.

## 2. Materials and Methods

### 2.1. Molecular Dynamics (MD) Simulation

Preliminary simulation of the behavior of the compounds in contact with the titanium surface was performed on the potential natural inhibitors. Monte Carlo (MC) and MD simulations were carried out, utilizing the Material Studio^®^ software 2023 (v23.1.0.3829 ×64). The objective was to gain a comprehensive understanding of how the molecules present in the green inhibitors interact with the surface of the oxide layer of titanium alloy. In particular, the Forcite model was employed for the simulation. The compounds being studied, including Caffeic acid, Ellagic acid, Gallic acid, Urolithine from pomegranate peel [37], and hydroxyphenyl chroman-4-one, 3,4-Dihydroxy cinnamic acid, Quercetin, and Kaemferol from tomato [38], were modeled to interact with the surface metal atoms of TiO_2_ (001) using the COMPASS III force field. Simulation on the algae extract was not performed due to the unsatisfactory results of the experimental tests (no inhibition effect). The chemical structure of the molecules of interest is listed in Table 1. TiO_2_ (001) was selected as the substrate due to its higher stabilization energy and densely packed structure [39,40,41]. The simulations were conducted with periodic boundary conditions and a vacuum slab thickness of 15 μm, within a simulation box measuring 70 Å × 70 Å × 100 Å. A time step of 1 ps (equivalent to 1000 fs) and a simulation duration of 10,000 ps were employed in the MD simulations, which were performed at 298 K in an NVT ensemble. A thermostat (Andersen approach) was utilized to maintain the simulation temperature constant at 298 K. The simulations offered insights into the adsorption mechanism and energy of these compounds on the surface of Ti alloy.

### 2.2. Preparation of Inhibitors

The pomegranate, tomato and algae were sourced from Shiraz, Bushehr and the Persian Gulf Sea, Iran. A straightforward, environmentally friendly, and water-based method was used to extract the chemical compounds from the pomegranate peel, tomato peel and algae. The extracts of pomegranate peel, algae and tomato peel were prepared using a similar method. For this purpose, after washing, 100 g of the materials was dried at a temperature of 80 °C for 12 h. The dried materials were then fully ground and pulverized using a laboratory grinder. Next, they were combined with 1 L of deionized water and heated at 70 °C for 8 h to extract the water-soluble compounds. Subsequently, the mixture was centrifuged at 3000 rpm for 20 min to completely eliminate any undissolved particles. Finally, the mixture was heated for 24 h at 80 °C to evaporate the water solvent and obtain the extract in powder form. To assess their inhibitory effects, approximately 700 ppm of each of the prepared materials were mixed in water in order to add this solution to the electrolyte. Therefore, due to the global low amounts of these inhibitors in the electrolyte, the pH of the electrolyte did not change significantly.

### 2.3. Sample Preparation and Corrosion Tests

An initial ingot of Ti grade 2 alloy was cut transversally with a disc cutter in lubricated conditions. As a result, square samples with a side of 15 mm and a thickness of no more than 3–4 mm were obtained. After cutting, the samples were ground and polished accordingly to a standard metallographic technique.

Then, the samples were washed with ultrasound to obtain an optimal surface finish free of impurities. Both potentiodynamic polarization (PDP) and electrochemical impedance spectroscopy (EIS) tests were conducted on the samples using a Gamry Interface 1010E potentiostat (Gamry Italia, Voghera (PV), Italy) in a three-electrode configuration using a Platinum electrode as a counter electrode, a calomel electrode as a reference electrode and the tested sample (1 cm^2^) as a working electrode. PDP tests were conducted between −2 V and +3 V with a scan speed of 1 mV/s. EIS tests were conducted with a sinusoidal perturbation of 10 mV over the frequency range of 100 kHz to 0.1 Hz. All the electrochemical tests were conducted after 30 min of OCP stabilization in two different electrolytes: 0.5 M NaOH and 3.5% NaCl aqueous solutions. Two different corrosive electrolytes were chosen in order to compare the behavior of the inhibitors in a chloride-containing environment and in an alkaline environment. The tests were firstly conducted in the solution without the inhibitor, as reference, and after the addition of 5% of the water-based solution containing the corrosion inhibitor (pomegranate, tomato and algae extract, obtained with the method described in Section 3.1). The results of the EIS were then fitted with a proper equivalent circuit using Z-View software (version 3.3). Corrosion potentials and corrosion currents were graphically extrapolated from PDP tests using the Tafel law, and then the data were employed to calculate the inhibition efficiency. All the corrosion tests were performed in triplicate in order to ensure reproducibility.

## 3. Results

### 3.1. Molecular Dynamic Simulation

**Figure 1:**
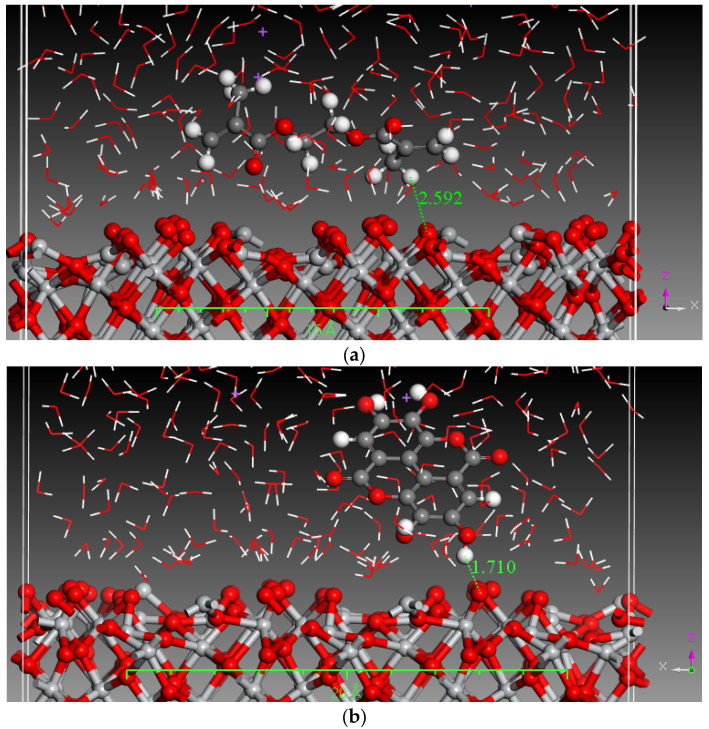

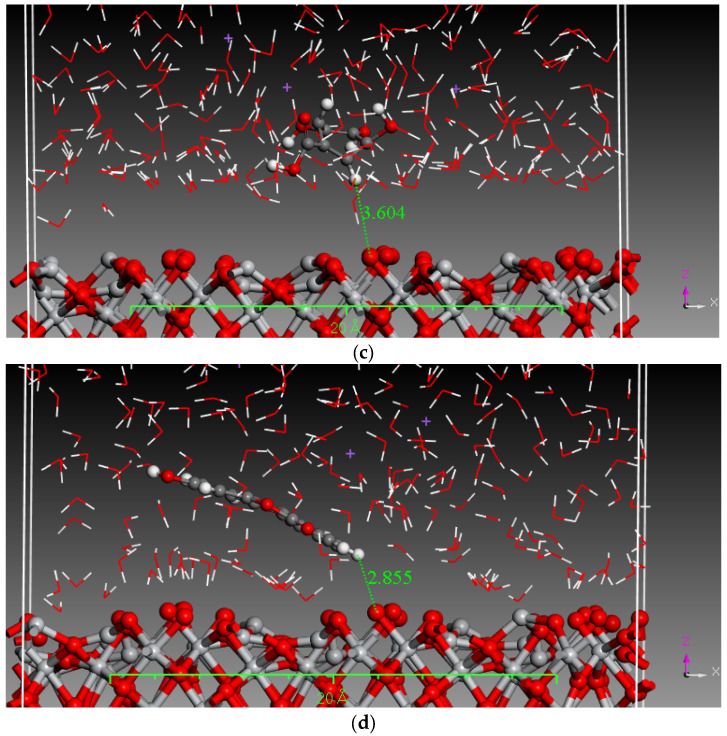
MD simulation of the constituent components of pomegranate peel extract in a 3.5% NaCl solution (**a**). Caffeic acid (**b**) Ellagic acid (**c**) Gallic acid and (**d**) Urolithine.

**Figure 2:**
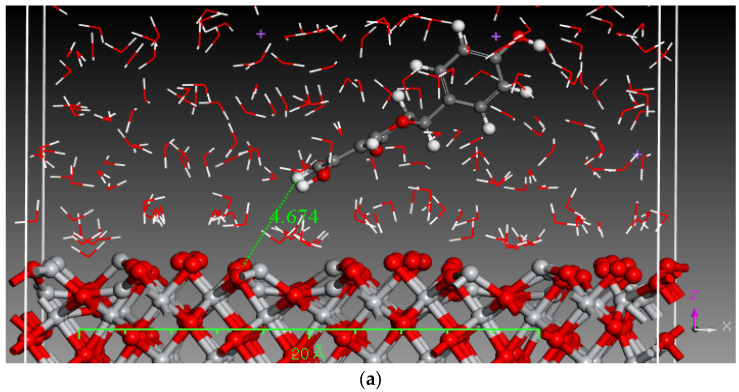

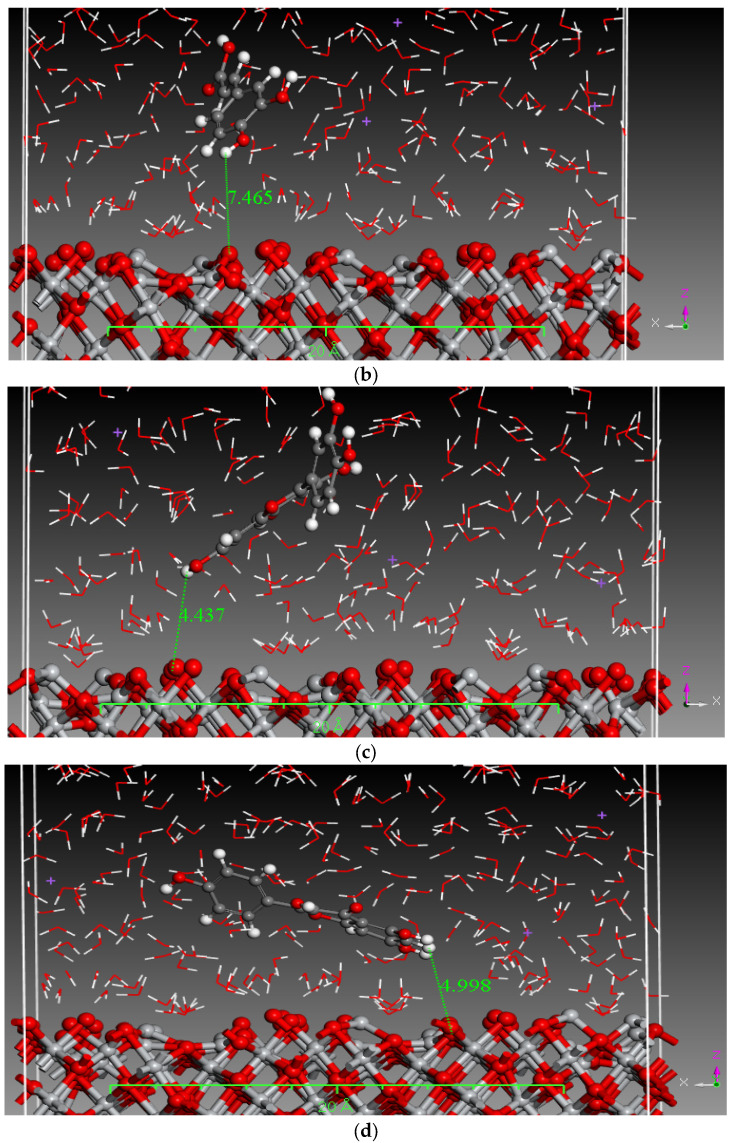
MD simulation of the constituent components of tomato extract in a 3.5% NaCl solution (**a**) hydroxyphenyl chroman-4-one (**b**) 3,4-Dihydroxy cinnamic acid (**c**) Quercetin (**d**) Kaemferol.

Monte Carlo (MC) and MD simulations were conducted to investigate the inhibition phenomenon on metal surfaces and to identify low-energy adsorption sites for the various compounds present in the inhibitors. These techniques have gained popularity for providing insights into the preferential adsorption of molecules on metal surfaces [21]. Figure 1 and Figure 2 present simulations of the adsorption orientation of different molecules on a titanium oxide (TiO_2_) surface. The results show that molecules exhibit a non-flat adsorption orientation on the TiO_2_ surface. After optimization, the estimated adsorption energies of different molecules on the TiO_2_ (001) surface and the corresponding adsorption geometries are listed in Table 2.

**Figure 3:**
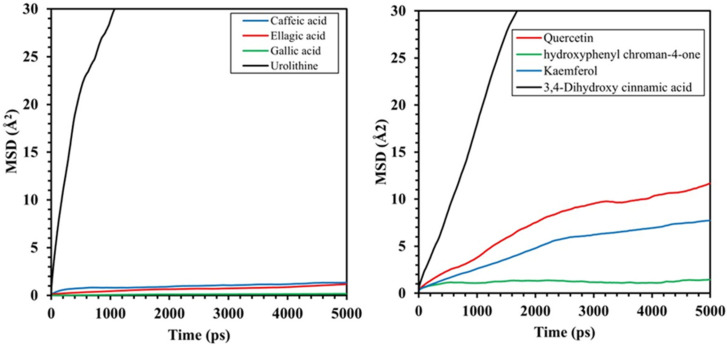
Mean Square Displacement (MSD) analysis.

Figure 3 shows the changes in the values of MSD for different molecules. The closer the slope is to the value of 1, the greater the ability of molecule movement in the electrolyte will be. Based on this, tomato extract components have the highest mobility capability in the electrolyte. The values of the diffusion coefficients for different molecules of pomegranate peel extract and tomato extract are listed in Table 2.

### 3.2. Open Circuit Potential (OCP) Measurement

**Figure 4:**
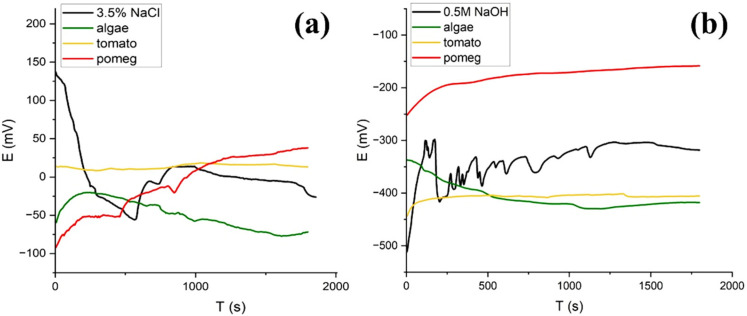
OCP decay analysis of the Ti samples in (**a**) 3.5% NaCl and (**b**) 0.5 M NaOH solution with/without natural inhibitors.

The OCP measurements were conducted to evaluate the thermodynamic stabilities of the grade 2 Ti samples in two environments: with and without inhibitors. Figure 4 shows the OCP measurements of grade 2 Ti samples in 3.5% NaCl (Figure 4a) and 0.5 M NaOH (Figure 4b) solutions with different natural inhibitors. In these figures it can be observed that the OCP values of the Ti samples in both solutions take about 1000 s to reach an indicative steady state. As shown in Figure 4a, by adding pomegranate to a 3.5% NaCl solution, the OCP of the Ti samples is shifted to the higher values. Conversely, the presence of algae in a sodium chloride solution led to a decrease in the OCP value of the Ti. In a 0.5 M NaOH solution, only pomegranate extract increases the OCP value of the Ti sample, from −319 mV to −158 mV. Both the tomato and algae decreased the OCP to −417 mV and −425 mV, respectively.

### 3.3. Electrochemical Impedance Spectroscopy (EIS)

**Figure 5:**
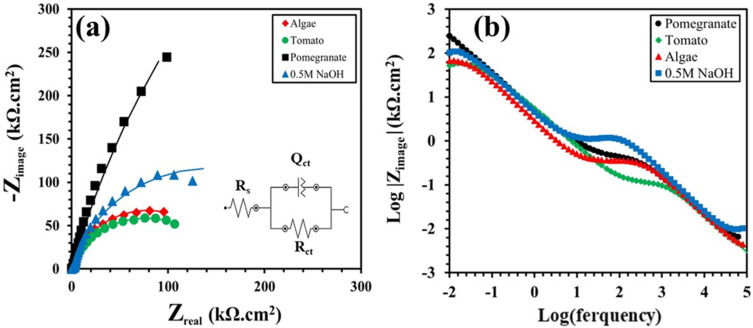
(**a**) Nyquist plot with the equivalent electrical circuit employed to fit the experimental data and (**b**) Bode modulus plot of the Ti grade 2 samples in 0.5 M NaOH solution with/without inhibitors.

**Figure 6:**
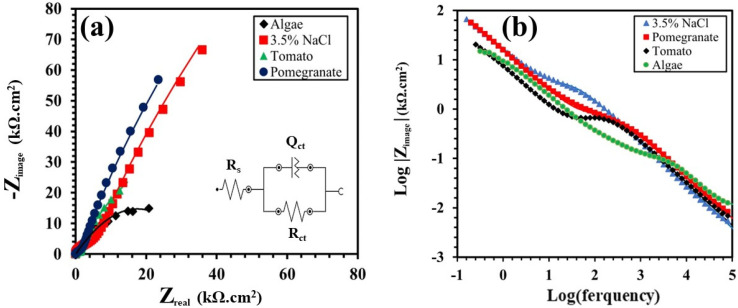
(**a**) Nyquist plot with the equivalent electrical circuit employed to fit the experimental data and (**b**) Bode modulus plot of the Ti samples in 3.5% NaCl solution with/without inhibitors.

Figure 5 and Figure 6 show the Nyquist and bode plots of the Ti grade 2 samples in 0.5 M NaOH and a 3.5% NaCl solution, with or without the natural inhibitors, whereas Table 3 and Table 4 report the results of the fitting of the experimental data. In these figures, the experimental and fitting data can be observed by the scattered dots and straight line, respectively.

Figure 5a displays the equivalent electrical circuit employed to fit the experimental data obtained by the EIS test. The circuit includes a solution resistance (*R_s_*) in series with a parallel capacitive loop charge transfer resistance and constant phase element (CPE) of the oxide layer formed on the surface (*R_ct_/Q_ct_*). The CPE is inserted into the circuit to serve as an alternative to the capacitor, aiming for a closer fitting under conditions where the frequency exponent is below 1.0 [46,47,48,49,50]. According to Figure 5a, the impedance at lower frequencies serves as an indicative measure of both the polarization resistance and the corrosion resistance. Therefore, the corrosion resistance values of the specimens are in the following order: pomegranate > 0.5 NaOH (reference) > tomato > algae. The charge transfer resistance (*R_ct_*) of the oxide layer is inversely proportional to the corrosion rate. According to Table 3, the *R_ct_* value of the Ti sample in a 0.5 M NaOH solution without any inhibitors is 2.82 × 10^5^ Ω.cm^−2^. By adding the pomegranate extract as an inhibitor, the *R_ct_* value was increased to 2.01 × 10^6^ Ω.cm^−2^. In other cases, it was observed that tomato extract and algae have a negative effect on the corrosion resistance of Ti samples in a NaOH solution, so that the *R_ct_* values decreased to 1.52 × 10^5^ and 1.56 × 10^5^ Ω.cm^−2^, respectively.

Bode impedance plots provide insights into different aspects of corrosion behavior. The high frequency range reflects the impact of surface defects caused by corrosion on the titanium oxide film. The middle frequency range correlates with the corrosion processes occurring within the film itself. Lastly, the low frequency range reveals information about corrosion at the interface between the metal and the oxide film. Referring to Figure 5b, it is observed that the low-frequency range of the algae exhibits significantly lower values in comparison to those of other inhibitors. This observation suggests that the poorly conductive TiO_2_ passive layer has been displaced.

Figure 6a displays the results of the EIS tests carried out on a Ti grade 2 sample in a 3.5% NaCl solution. In this case, it does not evidence a significant increase in corrosion resistance using the natural inhibitors. It can be observed that the use of pomegranate extract produces only a slight inhibition effect, especially in comparison with the results obtained in a 0.5 M NaOH solution. According to the equivalent electrical circuit (Figure 6b) and EIS parameters given in Table 4, the *R_ct_* value of the Ti sample in a 3.5% NaCl solution without any inhibitors, was increased from 2.82 × 10^5^ Ω.cm^−2^ to 2.01 × 10^6^ Ω.cm^−2^ by adding pomegranate extract. Inversely the addition of tomato and algae decreased the corrosion resistance of the Ti grade 2 alloy, so that the value of *R_ct_* significantly decreased to 92,413 and 39,450 Ω.cm^−2^, respectively. In Figure 6b, it is observed that the low-frequency range of the algae and tomato exhibit lower values compared to the samples tested with pomegranate extract and to the samples tested without any inhibitors.

### 3.4. Potentiodynamic Polarization (PDP)

**Figure 7:**
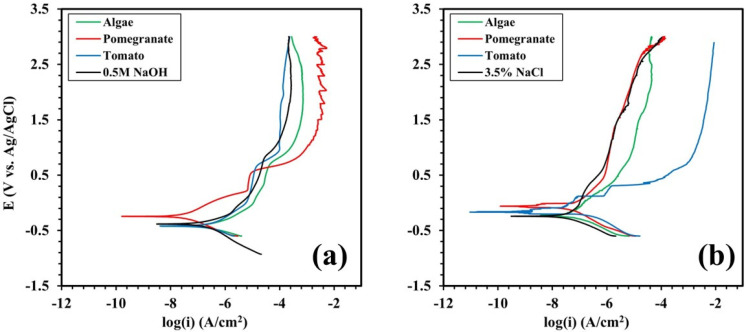
PDP curves of the Ti samples in (**a**) 0.5 M NaOH and (**b**) 3.5% NaCl solution with different inhibitors.

Figure 7 shows the potentiodynamic polarization curves of grade 2 Ti in two different solutions (0.5 M NaOH and 3.5% NaCl) adding different natural inhibitors.

Figure 7a shows the trends in the polarization curves in the case of titanium immersed in an aqueous solution 0.5 M NaOH + natural inhibitors. By considering the graph, it can be observed that the only curve that deviates from the solution without inhibitors (reference solution) is the curve of the pomegranate extract. Table 5 shows the corrosion parameters of the grade 2 Ti samples graphically extrapolated from PDP curves in 0.5 M NaOH, particularly the *i_corr_* and *E_corr_*. Graphical extrapolation was performed accordingly to the tafel method drawing the tangent to the cathodic and anodic branches of the curves. The presence of pomegranate extract obviously causes a shift in the *E_corr_* of NaOH from −383.7 to −244.08 mV. Also, a remarkable decrease in the *i_corr_* value of the sample tested in solution containing pomegranate extract can be observed, indicating a high inhibition effect.

Figure 7b illustrates the behavior of a grade 2 Ti sample in a 3.5% NaCl solution. From the graph, it can be observed that the natural inhibitors under consideration do not significantly increase the resistance of the material, as comparing the anodic branch of the different curves, it is evident that there is no improvement. Considering the corrosion potential there is a slight increase in this parameter employing tomato extract and a more significant increase in the presence of pomegranate extract. Globally, however, it can be stated that from PDP tests the inhibitors do not produce significant positive effects to the corrosion properties of grade 2 Ti in a 3.5% NaCl solution. Table 6 displays data graphically extrapolated from PDP tests in 3.5 NaCl. The presence of pomegranate extract causes a shift in the *E_corr_* from −254.57 to −82.40 mV. The *i_corr_* value of the sample tested with pomegranate extract in NaCl solution is 9.67 × 10^−7^ mA/mm^2^, which is the lowest between the different samples (and so the best in terms of corrosion resistance), but the differences are very small in comparison to the other tested samples. From the polarization curves it was possible to calculate the inhibition efficiency of the single inhibitors inserted in the different solutions. The efficiencies reported in Table 5 and Table 6 have been calculated using the following formula [51]:

(1) $$IE\%=\frac{\left(i_{0}-i\right)}{i_{0}}\times 100$$

where *IE%* is the inhibitors percentage efficiency, *i*_0_ is the current density in the case of the solution without inhibitors and *i* is the current density in the solution with the inhibitor of which the efficiency is to be calculated.

From the tables, it is possible to note that pomegranate extract has a good efficiency in all three different tested solutions whereas the effect of tomato extract and algae extract is controversial and, in any case, lower than the one of pomegranate extract.

## 4. Discussion

### 4.1. Simulations

The adsorption energy obtained from Monte Carlo (MC) simulations indicates that components of pomegranate peel extract have higher inhibition performance compared to tomato extract. The adsorption of Gallic acid molecules forms strong O-H chemical bonds with the TiO_2_ (001) surface, showcasing their effectiveness in protecting titanium against corrosion.

Additionally, the Mean Square Displacement (MSD) analysis is used to determine diffusion coefficient molecules, identifying different behavior of molecules in diffusion. The mean square displacement quantifies the movement of particles within a system and serves as a metric for characterizing molecular mobility. The formula for calculating MSD is expressed as [52]:

(2) $$\mathrm{MSD}=\langle {(r}_{i}\left(t\right)-r_{i}(0){)}^{2}\rangle$$

where r_i_ (0) and r_i_(t) denote the positions of particle *i* at the initial time and time t, respectively; the notation 〈〉 indicates the average value across all particles. The diffusion coefficient D for a particle can be determined by examining the slope of the MSD through the application of the Einstein relationship [52]:

(3) $$D=\frac{1}{6t}\underset{t\to \infty }{\lim}\langle |ri(t)-ri(0){|}^{2}\rangle$$

This relationship enables the calculation of the particle diffusion coefficient, providing insights into the dynamic behavior and mobility of particles within the system over time [52].

An overall investigation into the structural configuration, bonding energies, and permeability characteristics of diverse molecules suggests that within a 3.5% NaCl solution, Urolithin molecules exhibit the greatest efficacy among the components of pomegranate peel extract in obstructing functions. Likewise, within the constituents of the tomato extract, Quercetin molecules assume a pivotal role. In general, the higher bond energies of the constituents within pomegranate peel extract, coupled with their lower permeability coefficient relative to those found in tomato extract, suggest that the formation of a relatively stable molecular film on the titanium oxide surface by pomegranate peel extract may enable it to exert a more potent inhibitory effect.

According to the OCP measurements, the higher OCP values of the Ti sample in both solutions and with the presence of pomegranate extract, indicate the higher thermodynamic stability of the samples. The results from EIS and PDP tests indicate that these natural inhibitors exhibit varying degrees of efficacy, particularly in alkaline and saline solutions.

### 4.2. Efficacy of Natural Inhibitors

Pomegranate extract demonstrated a significant corrosion inhibition effect for Ti grade 2 in a 0.5 M NaOH solution. The increase in charge transfer resistance (*R_ct_*) indicates a substantial protective effect, likely due to the formation of a more stable oxide layer on the titanium surface. This finding aligns with previous studies that highlight the role of organic compounds in enhancing the stability of passive films on metals. For instance, it has been shown that polyphenols present in pomegranate can adsorb onto metal surfaces, forming a protective barrier that inhibits corrosion by reducing ion diffusion through the oxide layer [53].

In contrast, the addition of tomato extract and algae resulted in decreased *R_ct_* values, suggesting that these extracts may not only lack inhibitory properties but could potentially worsen corrosion processes. Research has indicated that certain organic compounds can disrupt protective oxide films rather than strengthening them [54]. The phytochemicals in tomato and algae may interact unfavorably with the titanium surface, leading to a compromised passive layer and increased corrosion susceptibility.

### 4.3. Corrosion Behavior in Saline Environments

When evaluating titanium’s corrosion behavior in a 3.5% NaCl solution, the effectiveness of pomegranate extract as an inhibitor was notably less pronounced compared to its performance in alkaline conditions. The slight increase in *R_ct_* with pomegranate extract highlights that while it provides some level of protection, its efficiency is significantly reduced in saline environments. This observation is consistent with the literature indicating that chloride ions can destabilize oxide layers on titanium, leading to increased susceptibility to localized corrosion [55].

The polarization curves further illustrate this trend; while there was a shift in corrosion potential (*E_corr_*) with pomegranate extract, the overall improvement in corrosion current density (*i_corr_*) was minimal. This suggests that while pomegranate extract has some inhibitive properties, its effectiveness is conditional upon specific environmental conditions, particularly the presence of aggressive ions like chloride. Previous studies have shown that although some natural extracts may enhance corrosion resistance under certain conditions, their performance can be compromised when exposed to aggressive electrolytes [56].

### 4.4. Mechanisms of Inhibition

The mechanisms by which pomegranate extract inhibits corrosion may involve both physical adsorption onto the titanium surface and chemical interactions with the oxide layer. The polyphenolic compounds found in pomegranate are known for their antioxidant properties and their ability to form complexes with metal ions [57]. This interaction may help to stabilize the passive layer on titanium by preventing the dissolution of metal ions into the electrolyte.

Conversely, the negative effects observed with tomato and algae extracts raise concerns regarding their application as corrosion inhibitors. The phytochemicals present in these extracts may not only fail to enhance protective mechanisms but could also introduce additional corrosive species or facilitate localized attack on the titanium surface. Understanding these interactions is crucial for developing effective strategies for enhancing the longevity of titanium implants.

## 5. Conclusions

In this study, the effects of three natural inhibitors (pomegranate, tomato and algae extract) on the corrosion behavior of grade 2 Ti in two different environments (0.5 M NaOH and 3.5% NaCl) was investigated. The main findings of the work can be summarized as follows:

- MD simulations revealed that components of pomegranate peel extract form strong O-H chemical bonds with the titanium oxide surface, indicating a robust inhibition capability. Additionally, MSD analysis highlighted differences in molecular mobility within the electrolyte, suggesting varied inhibitory potentials among the natural extracts tested.
- OCP decay and EIS tests evidence the promising properties of pomegranate extract as natural corrosion inhibitor for grade 2 titanium in 0.5 M NaOH solution. The presence of the inhibitor in fact produces a shift toward nobler potentials of the OCP and a remarkable increase in the polarization resistance.
- Potentiodynamic polarization tests confirmed the potential of pomegranate extract as a natural inhibitor, even if the effect under polarization is less pronounced than the effect observed at the equilibrium condition with EIS.
- The inhibition mechanism can involve both physical adsorption onto the titanium surface and chemical interactions with the oxide layer.
- In conclusion, it is advisable to choose pomegranate extract as a possible natural inhibitor to conduct further investigations in order to clarify better the mechanism of inhibition.

## Figures and Tables

**Table 1:** Chemical component information under investigation in this study.

Name	Chemical Structure	Ref.
Pomegranate peel extract		
Caffeic acid	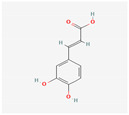	[37]
Ellagic acid	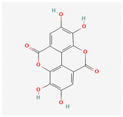	
Gallic acid	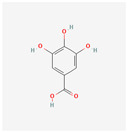	
Urolithine	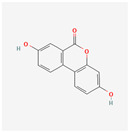	
Tomato extract		
hydroxyphenyl chroman-4-one	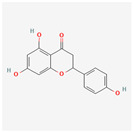	[42]
3,4-Dihydroxy cinnamic acid	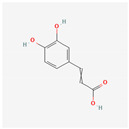	[43]
Quercetin	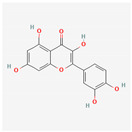	[44]
Kaemferol	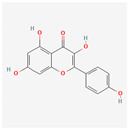	[45]

**Table 2:** Binding energy and diffusion coefficient of component in a 3.5% NaCl solution.

Components	Binding Energy (kcal)	Diffusion Coefficient D (A^2^/ps)	R^2^
Pomegranate peel extract			
Caffeic acid	−4.50 × 10^3^	2.424490 × 10^−5^	0.967
Ellagic acid	−1.81 × 10^4^	3.171714 × 10^−5^	0.945
Gallic acid	−1.91 × 10^4^	3.499181 × 10^−6^	0.988
Urolithine	−1.88 × 10^4^	3.904222 × 10^−4^	0921
Tomato extract			
Hydroxyphenyl chroman-4 one	−3.45 × 10^3^	1.409890 × 10^−4^	0.932
3,4-Dihydroxy cinnamic acid	−4.04 × 10^3^	6.265649 × 10^−4^	0.941
Quercetin	−4.06 × 10^3^	1.645273 × 10^−4^	0.962
Kaempferol	−4.02 × 10^3^	4.103204 × 10^−4^	0.933

**Table 3:** Results of the fitting of the experimental EIS data for Ti grade 2 in 0.5 M NaOH with/without inhibitors.

Sample	R_sol_ (Ω.cm^−2^)	R_ct_ (Ω.cm^−2^)	Q		χ (%)
			n	Y	
0.5 M NaOH	23.23 ± 0.5	(2.82 ± 0.02) × 10^5^	0.79	1.30 × 10^−5^	1.28
Pomegranate	22.72 ± 0.6	(2.01 ± 0.01) × 10^6^	0.85	3.51 × 10^−5^	1.93
Tomato	26.70 ± 0.4	(1.52 ± 0.03) × 10^5^	0.83	3.81 × 10^−5^	1.18
Algae	24.23 ± 0.8	(1.56 ± 0.02) × 10^5^	0.85	3.53 × 10^−6^	1.51

**Table 4:** Results of the fitting of the experimental EIS data for grade 2 Ti in 3.5% NaCl solution with/without inhibitors.

Sample	R_sol_ (Ω.cm^−2^)	R_ct_ (Ω.cm^−2^)	Q		χ (%)
			n	Y	
3.5% NaCl	46.35 ± 0.4	(8.14 ± 0.01) × 10^5^	0.81	1.30 × 10^−5^	1.39
Pomegranate	36.54 ± 0.5	(1.53 ± 0.02) × 10^6^	0.80	1.36 × 10^−5^	1.57
Tomato	54.62 ± 0.8	92,413 ± 110	0.84	2.61 × 10^−5^	1.11
Algae	31.14 ± 0.9	39,450 ± 240	0.83	1.53 × 10^−5^	1.80

**Table 5:** Corrosion current density, corrosion potential and IE% calculated from PDP performed on grade 2 Ti samples in 0.5 M NaOH.

Sample	E₀ (mV)	I₀ (mA/mm^2^)	IE%
0.5 M NaOH	−383.7	(1.12 ± 0.01) × 10^−6^	0
Algae	−421.38	(9.37 ± 0.01) × 10^−7^	16%
Tomato	−417.28	(5.71 ± 0.01) × 10^−7^	49%
Pomegranate	−244.08	(4.65 ± 0.01) × 10^−7^	58%

**Table 6:** Corrosion current density, corrosion potential and IE% calculated from PDP performed on grade 2 Ti samples in 3.5% NaCl.

Sample	E₀ (mV)	I₀ (mA/mm^2^)	IE%
3.5% NaCl	−254.57	(2.67 ± 0.01) × 10^−6^	0
Algae	−248.99	(4.04 ± 0.01) × 10^−6^	−52%
Tomato	−167.65	(1.01 ± 0.01) × 10^−6^	62%
Pomegranate	−82.40	(9.67 ± 0.01) × 10^−7^	64%

## Data Availability

The raw data supporting the conclusions of this article will be made available by the authors on request.
